# Variation in blubber thickness and histology metrics across the body topography of a false killer whale (*Pseudorca crassidens*)

**DOI:** 10.3389/fphys.2023.1001734

**Published:** 2023-04-03

**Authors:** Jana E. Phipps, Ilse Silva-Krott, Jamie Marchetti, Kristi L. West

**Affiliations:** ^1^ Health and Stranding Lab at Hawai‘i Institute of Marine Biology, University of Hawai‘i at Mānoa, Kāne‘ohe, HI, United States; ^2^ Human Nutrition Food and Animal Sciences, College of Tropical Agriculture and Human Resources, University of Hawai‘i at Mānoa, Honolulu, HI, United States; ^3^ Pacific Islands Regional Office, National Oceanic and Atmospheric Association, Honolulu, HI, United States

**Keywords:** false killer whale (*Pseudorca crassidens*), adipocyte, blubber, body condition, histology, health

## Abstract

Blubber is a multifunctional tissue essential to the survival of cetaceans. Histological assessment of blubber may be useful in determining odontocete nutritional state but a greater understanding of specific variation across the body is needed. We report on morphological variation of the blubber according to girth axes and sampling planes in a sub-adult male, bycaught false killer whale (*Pseudorca crassidens*) using metrics of blubber thickness (BT), adipocyte area (AA), and adipocyte index (AI). 48 full depth blubber samples were taken along 6 girth axes at 5 equidistant sampling points on both sides of the body. At these sampling locations BT was recorded, and AA and AI were determined for three distinct blubber layers. Linear mixed effect models were used to assess variation of the blubber across layers and body topography. BT was somewhat non-uniform across the body but was generally thicker in the dorsal region and thinner laterally. AA was greater cranially and AI was greater caudally. The middle and inner layer blubber showed significant differences dorsoventrally with larger AA and smaller AI in the ventral region of the body. Variation of the blubber metrics across the body are indicative of variable functions of the blubber within an individual. Due to the variability observed, we expect that AI of the dynamic inner layer blubber is most informative of overall body condition and that biopsy samples of the outer and middle blubber may still be useful in determining the nutritional status of live false killer whales.

## 1 Introduction

There are three distinct population segments (DPS) of false killer whales in Hawai‘i. The insular main Hawaiian Islands (MHI) population was listed as endangered under the endangered species act (ESA) in 2012 due to its small population size and observed population decline over the previous two decades (Baird, 2009; Reeves et al., 2009; Oleson et al., 2010; NOAA Fisheries, 2021). The most recent abundance estimate for the MHI DPS is 167 (Bradford et al., 2018). An ESA recovery plan and implementation strategy describes 19 threats to the MHI DPS. Threats of most concern include incidental take and inadequate regulatory mechanisms; however, reduced prey availability and compromised health are additional threats that are likely to impact population health and need to be addressed (NOAA Fisheries, 2021).

An important aspect of the recovery plan and implementation strategy is the monitoring of health. Body condition assessment is commonly used to relate population health to foraging success and food availability (Lockyer, 1986; Williams et al., 2013; Castrillon and Nash, 2020; Raverty et al., 2020). Although acknowledged as an important aspect of population and individual health assessment, there is little consensus on the best method for determination of body condition of live cetaceans and a wide range of direct and indirect measures have been used across species (Kershaw et al., 2017; Castrillon and Nash, 2020; Derous et al., 2020). Most methods focus on measures of blubber quantity; however, these methods can be complicated due to the heterogeneity of form and function of blubber within an individual and between species (Castrillon and Nash, 2020). In this study, measures of blubber quantity, using blubber thickness (BT) as well as blubber histology metrics as proxies, are considered in the context of better understanding false killer whale blubber complexity and body condition.

Blubber is a complex and specialized fat tissue characteristic of all marine mammals, and metrics are needed to determine energetic status and condition. Quantity of blubber is commonly used as an assessment of available energy storage however other tissue properties may be more meaningful due to the multifunctionality of the blubber in cetaceans. Blubber is made of densely packed adipocytes surrounded by collagenous fibers and is variable in the relative density of collagenous fibers, degree of vascularity, and size of adipocyte cells across blubber layer and body topography in a variety of cetacean species (Lockyer, 1986; Ahima and Flier, 2000; Koopman et al., 2002; Struntz et al., 2004; Iverson, 2009; Gómez-Campos et al., 2015). Furthermore, blubber morphology and function may be different among species and groups illustrating that an assessment of blubber quantity must be species specific to ensure meaningful results (Castrillon and Nash, 2020). Recent studies have used histological measurements of adipocyte area (AA) and adipocyte index (AI) of blubber to assess nutritional status in cetaceans (Castrillon et al., 2017; Druskat et al., 2019; Currie et al., 2021). AA is a measurement of the surface area of adipocyte cross sections in hematoxylin and eosin (H&E) stained blubber slides. It is commonly used as a proxy for blubber quantity in cetaceans (Castrillon and Nash, 2020; Struntz et al., 2004; Gómez-Campos et al., 2015). The AI was introduced as an alternative to measuring the more time-consuming AA metric in a study examining blubber biopsies from humpback whales taken at different times during the fasting season in a southern ocean population (Castrillon et al., 2017). While this study found that the two metrics, AA and AI, were correlated, AI has not previously been examined in a comprehensive way as a proxy for blubber quantity in odontocetes. We assessed both AA and AI and describe their relationship and utility in examining blubber quantity and nutritional status in the false killer whale.

In this study we establish baseline values of blubber quantity metrics according to morphology in a presumed healthy sub-adult male false killer whale and provide recommendations for the application of these metrics when assessing body condition in this species, including the use of biopsy samples collected from live false killer whales. Specific objectives included: 1) Examination of the relationship between AA, AI, and BT in an individual false killer whale; 2) Comparison of BT by girth, AA, and AI according to blubber layer and by girth; 3) Comparison of BT by body plane, AA, and AI according to blubber layer and by body plane; and 4) Assessment of the utility of BT, AA, and AI measurements when scoring the body condition of individual false killer whales.

## 2 Materials and methods

### 2.1 Sample collection

On 6 December 2019, a fishery interaction resulted in the mortality of a sub-adult male false killer whale. The National Oceanic and Atmospheric Association Fisheries Service Pacific Islands Region Observer Program recovered the carcass and stored it in an onboard freezer until the vessel returned to Honolulu the night of 18 December 2019. Upon arrival the animal was transported to the University of Hawai‘i Health and Stranding Lab and frozen until necropsy on 4 February 2020.

**FIGURE 1:**
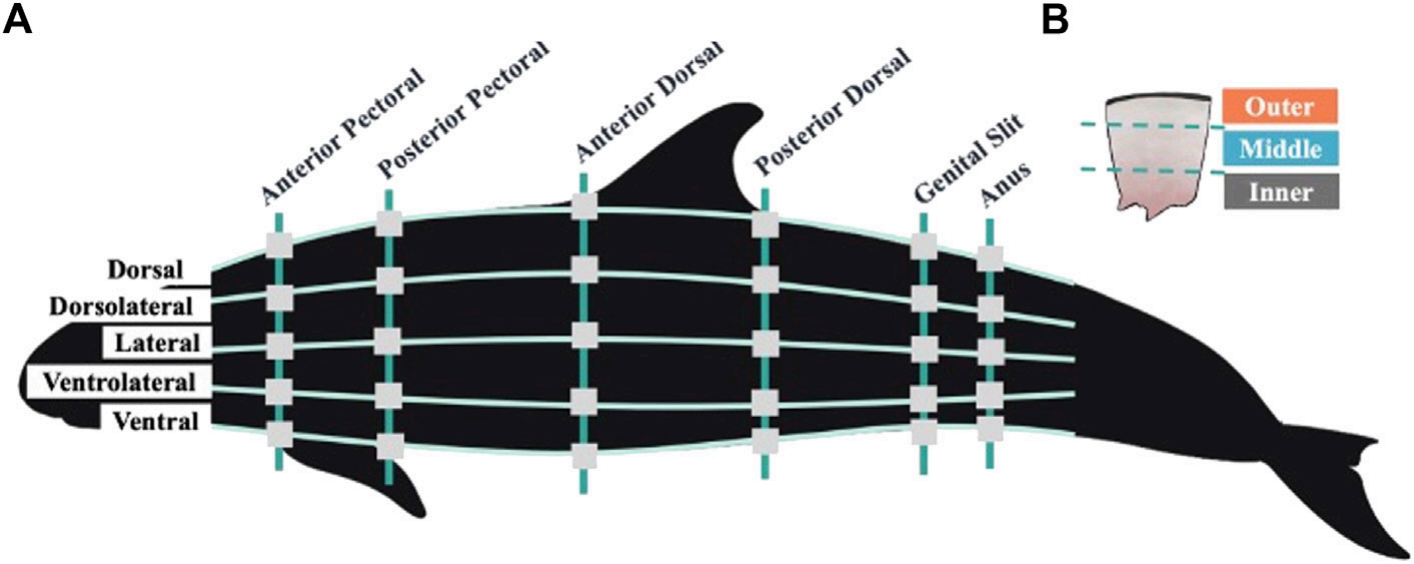
**(A)** Visual representation of blubber sampling locations across the left side of the false killer whale body. Vertical lines represent six anatomically identified girth axes, horizontal lines represent five equidistant sampling planes. Square gray boxes show the locations at which blubber was sampled during necropsy. While not illustrated here, the dorsolateral, lateral, and ventrolateral sampling planes were mirrored on the right side of the animal’s body at all girth axes resulting in a total of 48 sampled locations across the body topography. **(B)** Illustration of a single blubber sample and the three blubber layers. Illustrations created by Conner Humann and Jana Phipps using Procreate digital illustration app and Photoshop.

The individual appeared to be in good body condition, weighed 221 kg and had a total body length of 277 cm. Histopathological examination of the testes indicated the animal was immature at time of death. A full stomach was noted upon necropsy with intact and partially digested mahi mahi (*Coryphaena spp*.) fishes present. The false killer whale length and weight were compared to a growth curve based on repeated measures of 12 captive false killer whales (Kastelein et al., 2000). Based on length this individual weighed 19 kg less than expected however Kastelein et al. (2000) suggested that the growth curve equation overestimated weights of wild animals. Growth curve results and the absence of pathological findings indicative of poor nutritional status support the assumption that the bycaught false killer whale was in good body condition. An extensive blubber sampling protocol, including 48 sampled locations, was conducted during necropsy to generate baseline data on AA, AI, and BT across topographical regions of this presumed healthy false killer whale (Figure 1).

At the time of necropsy, blubber core samples were cut to encompass the full blubber layer from the epidermal skin layer to the muscle blubber boundary that is characterized by a thin layer of loose connective tissue. At each location sampled BT was recorded. Cross sections of the full blubber layer taken from blubber core samples were approximately 2 cm wide and 0.25 cm thick and were fixed in 10% buffered formalin at the time of sampling for histological processing. Blubber has a very high lipid content that protects the tissue from significant change due to freezing (Pond and Mattacks, 1985). Furthermore, the examination of the blubber using histology methods described in previous studies either included freezing as a consistent step in processing or potential changes were considered negligible to resulting findings (Castrillon et al., 2017; Koopman et al., 2002; McClelland et al., 2012).

Blubber samples were collected from eight points around six anatomically identified girth axes of the animal, resulting in a total of 48 sampling locations across the entire body topography (Figure 1). The girth axes included the anterior and posterior insertions of the pectoral fin, the anterior and posterior insertions of the dorsal fin, and the middle of the genital and anal slits. Previous odontocete blubber quantity studies suggest that locations cranial to the anterior pectoral fin may confound data regarding the energy storage function of blubber as the head contains acoustic fats which are distinct in function and morphology and not a focus in this investigation (Norris, 1968; Ackman et al., 1971; Koopman et al., 2002; Gómez-Campos et al., 2015). Equidistant sampling planes were identified along each of the anatomically identified girths. Sampling took place at the dorsal ridge and ventral most locations at each girth, at the midline on each side of the body, at a midpoint between the midline and dorsal, and midline and ventral locations, totaling eight locations. These were then categorized as five sampling planes to include the dorsal, dorsolateral, lateral, ventrolateral, and ventral planes (Figure 1).

Girth measurements and BT were also obtained using measuring tapes and electronic calipers at each location where blubber was sampled.

### 2.2 Histological preparation and imaging

Fixed blubber tissue samples were embedded in paraffin and the resulting blocks were cut into five-micron sections and mounted onto glass slides (Parlee et al., 2014; Castrillon et al., 2017). Slides were stained with H&E for histological analysis using standard protocols. All tissue embedding and slide processing was conducted at the Histology and Imaging Core Facility at the University of Hawai‘i, John A. Burns School of Medicine.

**FIGURE 2:**
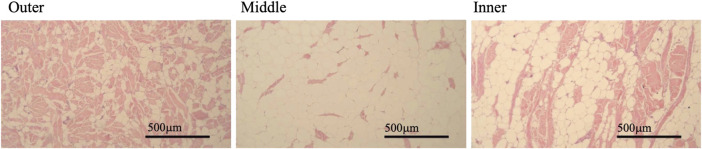
Example images of blubber from three visually identified layers in a single slide made from a full depth blubber sample. Blubber layer indicated by label above each image in panel. Pink stained areas of blubber indicates connective tissue stained, white unstained areas indicates adipocyte vacuolar space. Images taken by Jana Phipps using Jentopix Gryphax Arktur digital camera mounted on an Olympus BX-41 microscope at × 100 magnification.

Slides were viewed using an Olympus BX-41 microscope at ×100 magnification and grey scale images were captured using a Jentopix Gryphax Arktur digital camera. From each slide three images were taken of each of the three stratified layers of blubber: outer, middle, and inner layers (Figure 2). Based on descriptions and imaging in previous studies of bottlenose (*Tursiops truncatus*) and striped dolphins (*Stenella coeruleoalba*) each layer was visually identified by the relative amount of structural and connective tissue observed through the depth of the blubber (Montie et al., 2008; Gómez-Campos et al., 2015). The outermost layer just below the dermis appeared darker by H&E stain due to more connective tissue; dermal tissue was also visible adjacent to the outer blubber layer. The middle blubber layer had characteristically lighter H&E staining and adipocytes were more evenly distributed in comparison to the outer and inner layers (Figure 2). The innermost layer had qualitatively more variable cell size and structure, the H&E stain was moderate in intensity and this layer was directly adjacent to the loose connective tissue layer that separates blubber from muscle (Figure 2) (Struntz et al., 2004; Montie et al., 2008; Gómez-Campos et al., 2015). Nine images from each slide were used in image analysis of AA and AI.

### 2.3 Image analysis

#### 2.3.1 Adipocyte area (AA)

Using previously described methods open source Adiposoft Image Analysis software was used in the FIJI (ImageJ) interface to measure a minimum of 100 adipocytes per image (Galarraga et al., 2012; Schindelin et al., 2012; Parlee et al., 2014; Castrillon et al., 2017). The Adiposoft software output was manually corrected to ensure the quality of overall estimate of adipocyte size. Manual correction included the deletion of incorrectly identified cells as well as the identification of cells missed in the processing of the image. All cells measured in an image were used to determine an average AA for that image. This average AA measurement was then used for data analysis.

#### 2.3.2 Adipocyte index (AI)

In addition to the more commonly used AA metric we also assessed the recently described AI measurement that utilizes the threshold tool in FIJI (ImageJ) to determine a ratio value of intervacuolar space to the lipid filled adipocyte vacuolar space, estimating the relative lipid storage of the tissue sampled (Shapiro and Stockman, 2001; Ferreira and Rasband, 2012; Schindelin et al., 2012; Castrillon et al., 2017).

Gray scale images were uploaded into ImageJ and the threshold tool was set to darken all but the vacuolar space of the adipocyte at the discretion of the analyst. In Castrillon et al., 2017 a constant threshold value was set for the humpback whale blubber, however, in this study the threshold value was manually adjusted to best fit for each image. This step was taken due to the variability of stain intensity with blubber sample depth (Montie et al., 2008; Gómez-Campos et al., 2015). Once a binary image was produced using the threshold tool the number of black and white pixels were individually measured; black pixels represented connective tissue and white pixels represented adipocyte vacuolar space. A ratio of black:white pixels was calculated that represented the energy reserve exhibited in the image, a larger AI was suggestive of a sample with less relative lipid energy stored, a smaller AI was suggestive of a larger relative amount of lipid energy stored.

### 2.4 Data analysis

AA, AI, and BT data were evaluated for normal distribution and homogeneity of variance using Kolmogorov-Smirnoff goodness of fit and Levene’s test for equality of variance. When all sampled layers were combined into a single sample set the assumptions of normality and homogeneity were not met for AA and AI values even after transforming the data. Potential relationships between AA, AI, and BT were evaluated using Spearman’s method of correlation.

Kruskal–Wallis one-way analysis of variance and Dunn’s multiple comparisons for non-parametric data were used to assess differences in AA and AI metrics across blubber layers. Linear mixed effect models with Tukey’s multiple comparisons were used to examine the variability of AA and AI across girth axes and sampling planes by individual blubber layer because of the distinct differences in values observed between each layer.

Mixed effect models were used to assess the effect of girth axes and sampling plane on the AA, AI, and BT measurements. To assess the effect of girth axis it was identified as the fixed effect in the model and sampling plane was designated as the random effect. The random and fixed effects were reversed to assess the effect of sampling plane. The mixed model method allowed for one factor of sampling, either girth axis or sampling plane, to be the focus of the analysis while variability from the other or from an interaction of effects was discounted by being categorized as a random effect (Bates, 2007). All statistical analyses were performed using R Studio open-source software environment, mixed effects models were run using the package lme4, standard error calculated using the package psych. Figures were created in R Studio using ggpubr, ggplot2, and ggsci packages (v3.6.2; Rstudio Team, 2020).

## 3 Results

### 3.1 Blubber thickness (BT)

**FIGURE 3:**
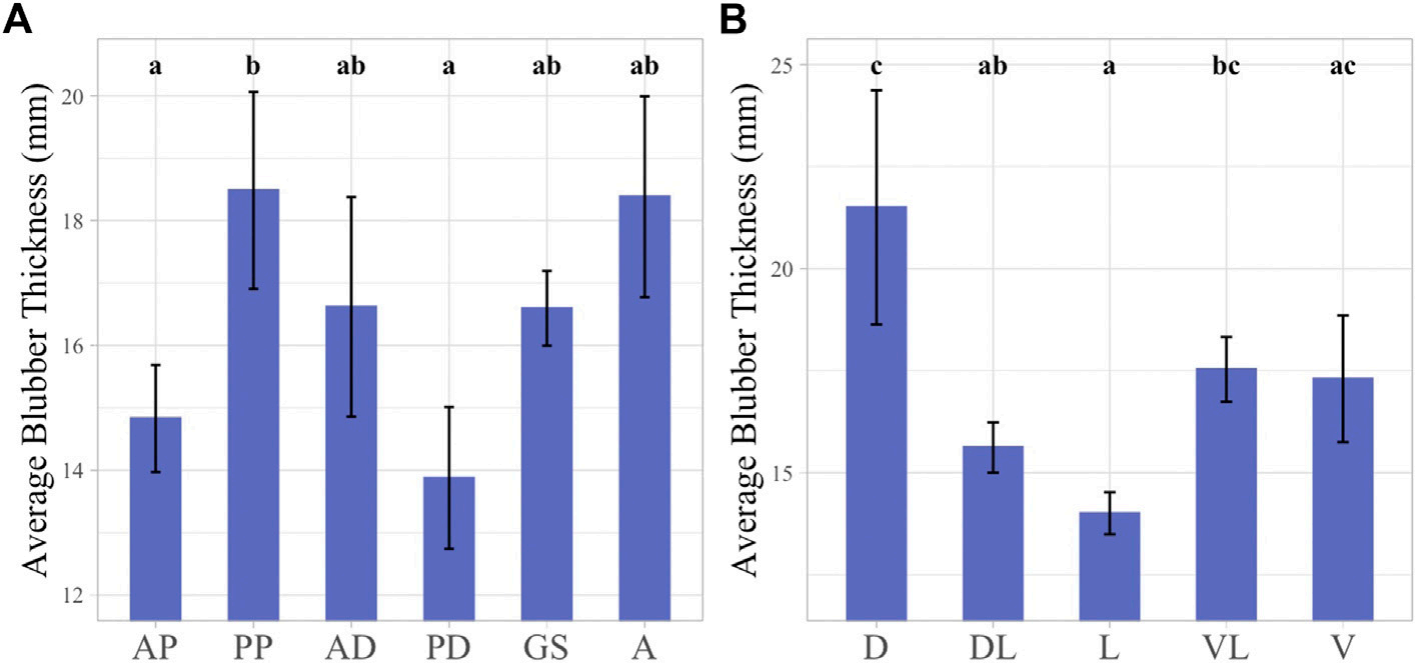
Linear mixed effect models illustrating average BT across girth axes **(A)** and sampling planes **(B)**. Girth axes labeled with 1-2 letter identifier, “AP” = Anterior Pectoral, “PP” = Posterior Pectoral, “AD” = Anterior Dorsal, “PD” = Posterior Dorsal, “GS” = Genital slit, “A” = Anus. Sampling planes labeled with 1-2 letter identifier, “D” = Dorsal, “DL” = Dorsolateral, “L” = Lateral, “VL” = Ventrolateral, “V” = Ventral. Significant differences between factors illustrated by lowercase letters above each bar graph. Groups with any shared letters are not significantly different to each other. Error bars represent standard error.

BT measurements were recorded for all 48 sampled locations and the average thickness among all sampling locations was 16.57 ± 0.56 mm (range: 11.02–28.52 mm). BT was examined across girth axes in the individual false killer whale and did not follow a clear pattern. The smallest average BT was found at the anterior pectoral and posterior dorsal girth axes, averaged 14.83 ± 1.76 mm and 13.9 ± 1.14 mm respectively, and were not significantly different from one another. The largest average BT was observed in the posterior pectoral girth axis which averaged 18.48 mm ± 1.58 and was significantly different from both the anterior pectoral and posterior dorsal girth axes. The largest variation within individual girth axes was observed at the posterior dorsal girth axis where BT ranged between 11.02 and 28.52 mm with large variation also evident at the anal girth axis (Figure 3A).

BT was also examined according to sampling planes. The highest average thickness was observed in the dorsal plane, 21.5 ± 2.87 mm, and this plane was significantly different from all other body planes except for the ventral and ventrolateral planes. The dorsal plane also exhibited the greatest variation in BT measurements among the body planes examined, ranging between 13.68 and 28.52 mm. The smallest average BT value was observed at the lateral plane measuring 14.06 ± 0.52 mm, and this was significantly different from the dorsal and ventrolateral planes (Figure 3B).

When looking at the BT data across the whole body of the individual the location with the largest BT measurement was at the posterior pectoral girth in the dorsal plane, with a measure of 28.52 mm and the smallest BT was located at the posterior dorsal girth in the lateral plane with a measure of 11.02 mm.

### 3.2 AA and AI comparison by layer

**FIGURE 4:**
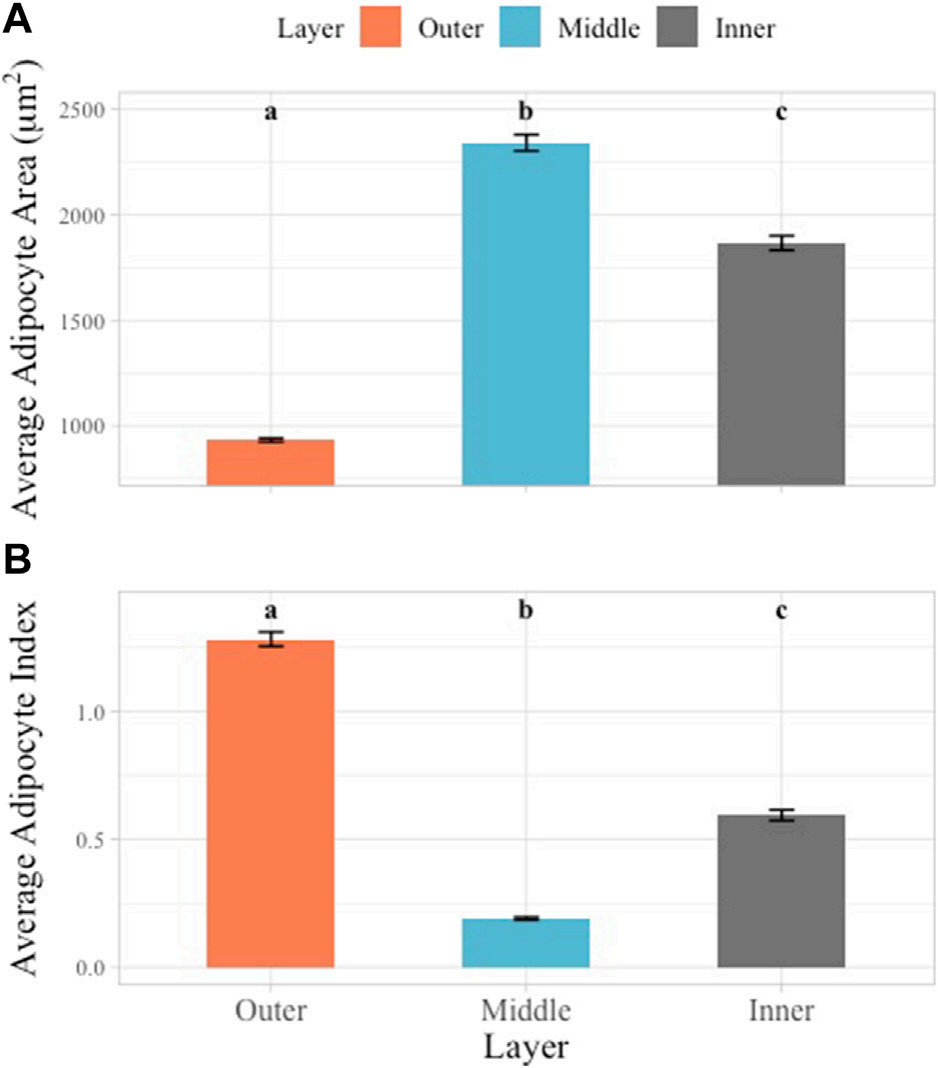
Multiple comparisons of average AA and AI across layers. Blubber layer represented by unique color. Multiple comparisons showed significant differences between all layers in AA **(A)** and AI **(B)** data. Significant differences illustrated using lowercase letters above each bar graph. Error bars represent standard error.

AA and AI were examined according to blubber layer. AA and AI both varied significantly between blubber layers (*p* < 0.05). The outer layer adipocytes had the smallest average AA (933.10 ± 8.92 μm^2^, range: 663.84–1,208.93 μm^2^), the middle layer the largest average AA (2,342.04 ± 38.53 μm^2^, range: 1,503.35–1914.59 μm^2^) and the inner layer an intermediate average AA (1866.67 ± 34.47 μm^2^, range: 1,001.73–2,922.51 μm^2^) (Figure 4A). An inverse relationship was observed in the AI values, with the largest AI observed in the outer layer (1.27 ± 0.03, range: 0.51–1.63), smallest in the middle layer (0.19 ± 0.01, range: 0.08–0.38), and intermediate in the inner layer (0.59 ± 0.02, range: 0.17–1.25) (Figure 4B).

### 3.3 Relationship between AA, AI, and BT

**FIGURE 5:**
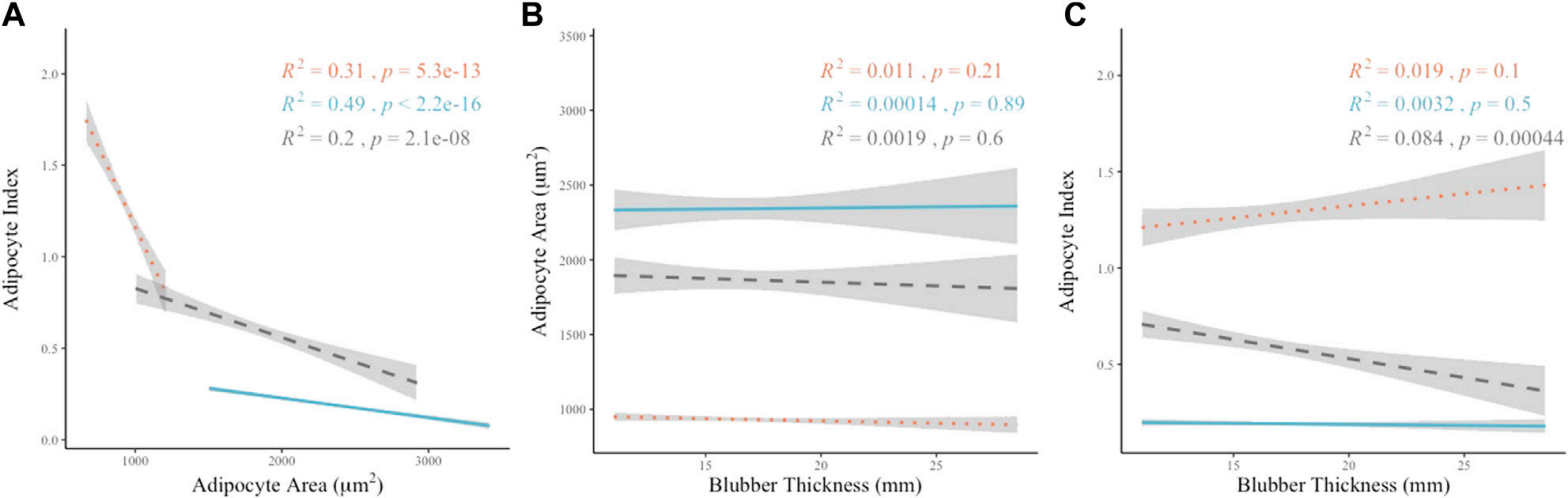
Linear correlation relationships between metrics of AA, AI and BT. Blubber layer identified by color and line shape. Outer layer data shown as orange dotted line, middle layer shown as blue solid line, inner layer shown as gray dashed line. **(A)** Linear correlations between AA and AI by Pearson’s method of correlation (inner *R* ^2^ = 0.2, *p* < 0.01; middle *R* ^2^ = 0.47, *p* < 0.01; outer *R* ^2^ = 0.32, *p* < 0.01). **(B)** Linear correlations between AA and BT by Pearson’s method of correlation (inner *R* ^2^ = 0.0019, *p* = 0.6; middle *R* ^2^ = 0.00014, *p* = 0.89; outer *R* ^2^ = 0.011, *p* = 0.21). **(C)** Linear correlations between AI and BT by Pearson’s method of correlation (inner *R* ^2^ = 0.084, *p* = 0.00044; middle *R* ^2^ = 0.0023, *p* = 0.57; outer *R* ^2^ = 0.015, *p* = 0.14).

A significant and strong linear correlation was observed between the AA and AI values (rs = −0.875, *p* < 0.01) when compared among all sampling locations (Figure 5A). The overall relationship between AA and AI indicates distinct groupings by layer and a stronger correlation between AA and AI when data from all sampling locations is combined as opposed to the relationship between AA and AI on an individual layer basis (Figure 5A). When considering the individual layers separately, the middle layer was most strongly correlated (*R* ^2^ = 0.49, *p* < 0.01), the inner layer weakly correlated (*R* ^2^ = 0.2, *p* < 0.01) and the outer layer of intermediate correlation strength (*R* ^2^ = 0.31, *p* < 0.01).

BT at each sampling location was also assessed for correlation with AA and AI on an individual layer basis. The correlations between BT and either AA or AI were not significant except for the relationship between inner layer AI and BT (*p* = 0.0004). *R* ^2^ values were weak in all cases when comparing BT to either AA or AI (Figures 5B, C).

### 3.4 Topographical variation in AA and AI across girth axes

#### 3.4.1 Outer layer AA and AI

**FIGURE 6:**
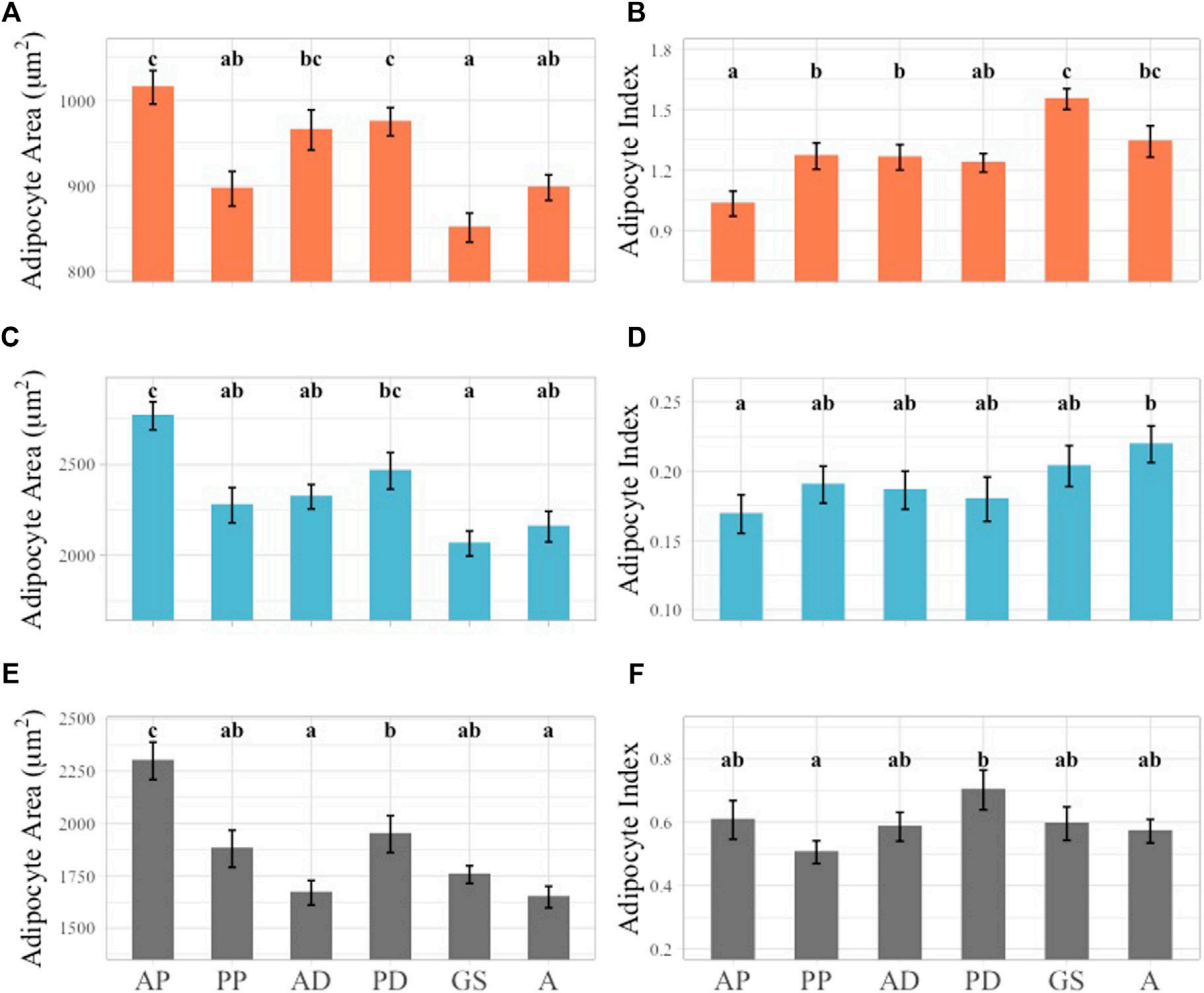
Linear mixed effect model of average AA and AI across girth axes subset by layer. Outer layer data shown in orange at top row of figure panel, middle layer shown in blue in middle row of figure panel, inner layer shown in gray in bottom row of figure panel. Significant differences identified by lower case letters above each bar graph. Groups with any shared letters are not significantly different to each other. Error bars represent standard error. **(A)** Outer layer AA**, (B)** Outer Layer AI, **(C)** Middle layer AA, **(D)** Middle Layer AI, **(E)** Inner layer AA, **(F)** Inner Layer AI. Girth axes for all plots shown using one to two letter identifier along *X*-axis of inner layer Panel 6E, F, “AP” = Anterior Pectoral, “PP” = Postersior Pectoral, “AD” = Anterior Dorsal, “PD” = Posterior Dorsal, “GS” = Genital slit, “A” = Anus.

Across girth axes AA and AI generally decreased and increased respectively from the cranial to caudal regions of the animal (Figures 6A, B). When the outer layer AA data was considered, the largest average AA was observed at the anterior pectoral axis and values were significantly different from all other girth axes except for anterior and posterior dorsal girth axes (Figure 6A). The smallest average AA was observed in the genital slit girth axis, but this was only significantly different from the anterior pectoral, anterior dorsal, and posterior dorsal girth AA values (Figure 6A). In the outer layer AI data, the smallest average AI value was observed in the anterior pectoral axis, and it was significantly different from the average AI values of all girth axes except for the posterior dorsal girth axis. The largest AI value was observed at the genital slit girth axis and was significantly different from all other axes apart from the anal girth axis (Figure 6B).

#### 3.4.2 Middle layer AA and AI

In the middle layer a general trend of larger AA and smaller AI cranially and smaller AA and larger AI caudally was observed. However, the general trend observed in the outer blubber layer was stronger in terms of statistical significance (Figures 6A–D). In the middle layer AA data, the largest average AA was observed at the anterior pectoral girth axis, and this was significantly different when compared to all the girth axes examined except for the posterior dorsal axis. The smallest average AA was observed at the genital slit axis but was only significantly different from the anterior pectoral and posterior dorsal girth axes (Figure 6C). Fewer significant differences were observed within the AI dataset when compared to the AA values. The largest average AI was observed at the anal slit girth axis and was only significantly different from the anterior pectoral girth axis (Figure 6D).

#### 3.4.3 Inner layer AA and AI

The general trend of decreasing AA from the cranial to caudal regions in the outer and middle blubber layers was also observed in the inner layer AA but not the corresponding increase in AI (Figures 6E, F). The largest average inner layer AA was observed in the anterior pectoral girth axis, and it was significantly different from all other girth axes measured. The anus girth had the smallest average AA value, but this girth axis was only significantly different from the anterior pectoral and posterior dorsal girth axes (Figure 6E). In the inner layer the largest average AI value was observed in the posterior dorsal girth axis and the smallest in the posterior pectoral axis, which was different from the general trend observed in the outer and middle blubber layers. The only significant differences between inner layer AI girths were observed between the posterior dorsal and posterior pectoral axes (Figure 6F).

### 3.5 Topographical variation in AA and AI across sampling planes

#### 3.5.1 Outer layer AA and AI

**FIGURE 7:**
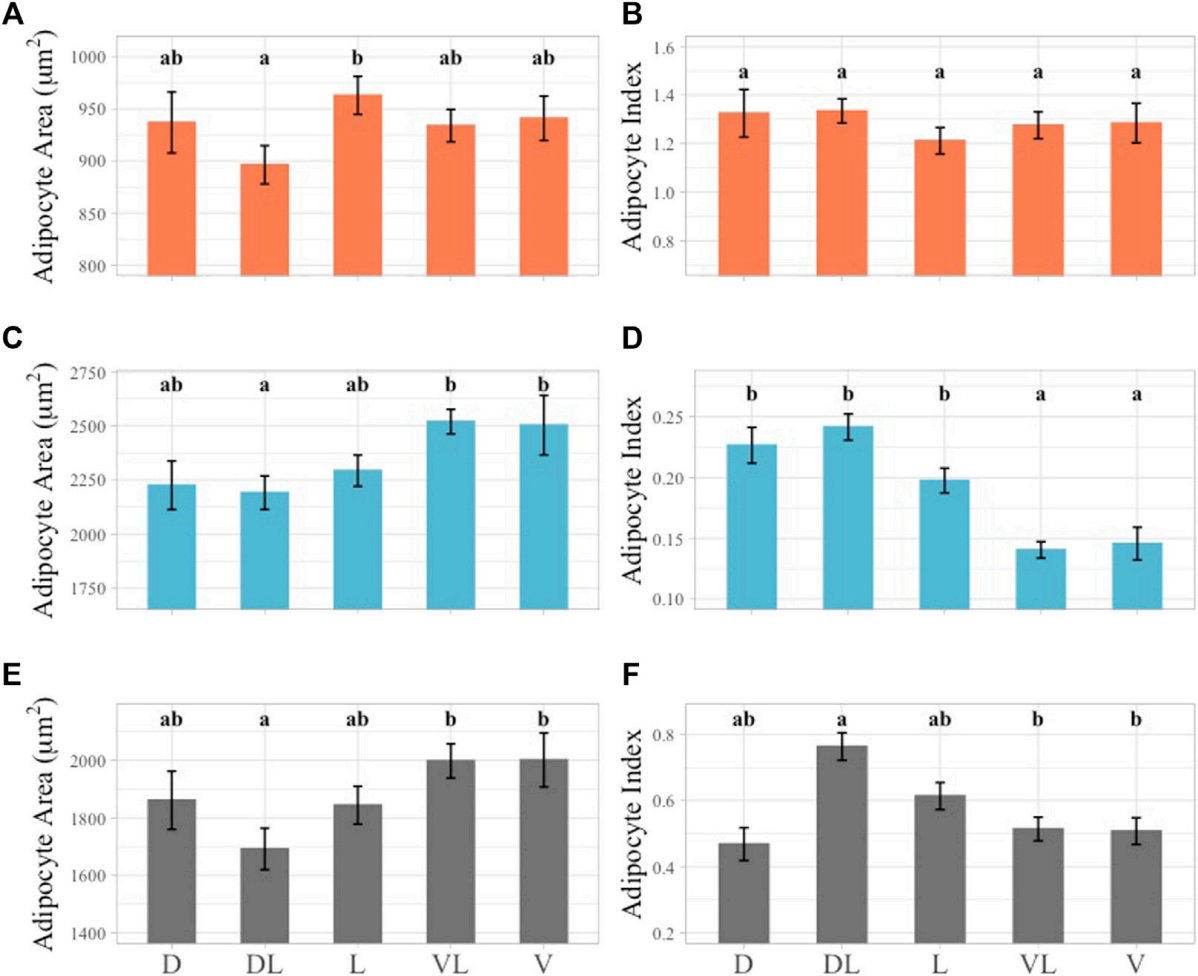
Linear mixed effect model of average AA and AI across sampling planes subset by layer. Outer layer data shown in orange at top row of figure panel, middle layer shown in blue in middle row of figure panel, inner layer shown in gray in bottom row of figure panel. Significant differences identified by lower case letters above each bar graph. Groups with any shared letters are not significantly different to each other. Error bars represent standard error. **(A)** Outer layer AA, **(B)** Outer Layer AI, **(C)** Middle layer AA, **(D)** Middle Layer AI, **(E)** Inner layer AA, **(F)** Inner Layer AI. Sampling plane for all plots shown using 1-2 letter identifier along *X*-axis of inner layer Panel 7E, F, “D” = Dorsal, “DL” = Dorsolateral, “L” = Lateral, “VL” = Ventrolateral, “V” = Ventral.

The largest average outer layer AA was observed in the lateral plane and the smallest in the dorsolateral plane and these two planes were significantly different from each other (Figure 7A). In the outer layer AI values, no significant differences were observed, with the highest average AI in the dorsolateral plane and the lowest in the lateral plane (Figure 7B).

#### 3.5.2 Middle layer AA and AI

The largest AA value observed in the middle blubber layer was in the ventrolateral plane and this was not significantly different from any other plane except for the dorsolateral plane. The dorsolateral plane had the smallest average AA value and was also significantly different from the ventral plane (Figure 7C). The largest AI value in the middle blubber layer was observed in the dorsolateral plane and was significantly different from the ventral and ventrolateral planes. The smallest average AI was observed in the ventrolateral plane and was significantly different from all the sampling planes except for the ventral plane (Figure 7D).

#### 3.5.3 Inner layer AA and AI

In the inner layer AA data, the smallest average AA was observed in the dorsolateral plane and this plane was also significantly different from the ventral and ventrolateral planes (Figure 7E). In the inner layer AI values, an inverse relationship to AA was observed (Figure 7F). The inner and middle layer AA and AI values indicated that dorsolateral planes were significantly different from the ventral and ventrolateral planes (Figure 7).

## 4 Discussion

This is the first study to our knowledge that links BT measurements to blubber histology metrics at the same body locations to assess the relationship among these metrics in cetaceans. Previous studies have considered BT across topography and its relation to other morphological metrics including length and mass, biochemical metrics such as lipid content and protein content, as well as studies relating thickness to measures of AA (Lockyer et al., 1985; Koopman et al., 2002; Struntz et al., 2004; Dunkin et al., 2005; Miller et al., 2011; Gómez-Campos et al., 2015; Irvine et al., 2017; Kastelein et al., 2018). We add to this body of knowledge with analysis of AA and AI from more topographical locations than previously assessed in an individual odontocete. Changes in AA and AI across the body and between blubber layers support region specific functions of blubber that vary by blubber layer and contribute to informing the potential application of these metrics as body condition indices.

### 4.1 BT across body topography

Direct measurement of BT at 48 locations along six girth axes and five body planes of a false killer whale that was presumed in good body condition was compared to histological blubber metrics at the same locations. Average BT varied across the girth axes and sampling planes of the body. The largest averages of BT were observed in the dorsal plane and the posterior pectoral girth axis, and the smallest were in the lateral plane and posterior dorsal girth axis with up to a 62% difference in thickness (Figure 3). Non-uniformity in BT across the body has been documented in several marine mammals (Lockyer, 1986; Doidge, 1990; Koopman, 1998; Koopman et al., 2002; Hamilton et al., 2004; Caon et al., 2007; Noren et al., 2015; Noren et al., 2021). This variation supports the diverse functions of the blubber across the body. The thoracic-abdominal region is important to insulation and short-term energy reserve and the caudal region is more important to locomotion and heat dissipation in harbor porpoises (*Phocoena phocoena*) (Koopman, 1998; Koopman et al., 2002). In both harbor porpoises and franciscanas (*Pontipora blainvillei*) greater variability across sampling planes in the caudal region of the body is attributed to the importance of keel stability for movement (Hamilton et al., 2004; Caon et al., 2007). We found the highest variability in BT in the anal girth suggesting a similar function in the false killer whale (Figure 3).

While BT variation within the body of odontocetes is well documented, there is less support for a correlation with body condition. BT has been used as an indicator of overall body condition in many cetaceans, however it appears to be more suited to determination of baleen whale health in comparison to odontocete body condition and nutritional status (Kershaw et al., 2018; Castrillon and Nash, 2020; Derous et al., 2020). In sperm whales (*Physeter macrocephalus*), franciscanas, harbor porpoises, and beluga whales (*Delphinapterus leucas*), BT was not indicative of nutritional or reproductive state suggesting it is not a good indicator of body condition (Read, 1990; Evans et al., 2003; Caon et al., 2007; Larrat and Lair, 2022). Smaller odontocetes may maintain a more consistent BT because of the ability to feed often and the importance of thermoregulation for a smaller body size (Gearty et al., 2018; Kershaw et al., 2018). The relationship between the BT metric and the histology metrics AA and AI were not correlated in this study except for the inner blubber AI and BT (Figure 5C). These results suggest that BT is not a strong indicator of nutritional or reproductive status in false killer whales with the caveat that our data are based on examination of one sub-adult animal.

### 4.2 Stratification of the blubber

Blubber histology metrics indicated clear morphological stratification of the three sampled blubber layers consistent with histological identification of layers (Figure 4). Findings aligned with previous studies in other cetacean species where the different layers were visually identifiable based on H&E stain intensity (Montie et al., 2008; Gómez-Campos et al., 2015; Castrillon and Nash, 2020). In South Africa, adipocyte cell size, number, and stain density were different among blubber layers in Indo-Pacific bottlenose dolphins (*Tursiops aduncus*) and common dolphins (*Delphinus delphis*) but not in Indian Ocean humpback dolphins (*Sousa plumbea*) as blubber stratification was not observed in this species (Roussouw et al., 2022). In this study, the false killer whale outer layer had the smallest cell size and the largest AI data, indicative of increased connective tissue in this layer. The middle layer had the largest average AA and smallest AI, and the inner layer was intermediate in both AA and AI data (Figure 4). This relative comparison in a false killer whale followed the same pattern as previously described in striped dolphins, Indo-Pacific bottlenose dolphins, and common dolphins (Gómez-Campos et al., 2015; Roussouw et al., 2022). Regarding striped dolphins, it was hypothesized that these differences among layers were related to variable function of blubber (Gómez-Campos et al., 2015).

### 4.3 Histological metrics across the body topography

Blubber samples from six girths axes and five body planes exhibited variation in the blubber histology indices of AA and AI across body regions (Figures 6, 7). AA and AI variation was assessed by layer due to the stratified structure of the blubber that was observed across the entire body of the animal. Larger average AA values were seen in anterior most girth axis, the anterior pectoral axis, and the posterior dorsal girth axis as well as the two most ventral sampling planes. AI values were higher towards the caudal end of the body and in the dorsal and dorsolateral planes, however, AI differences were not significant in the outer blubber layer across all girth axes and planes.

Regional variability in the blubber has been described from previous studies that have used blubber histology metrics, as well as morphology and biochemical methods to assess differences in the blubber across the body in a range of cetaceans including striped dolphins, harbor porpoises, short-finned pilot whales (*Globicephala macrorhynchus*), common dolphins, fin (*Balaenoptera physalus*), sei (*Balaenoptera borealis*), and minke whales (*Balaenoptera acutorostrata*) (Lockyer et al., 1985; Koopman, 1998; Tornero et al., 2004; Christiansen et al., 2013; Gómez-Campos et al., 2015; Noren et al., 2021). The high AA and low AI values in the anterio-ventral body region of this false killer whale with good nutritional status are consistent with lipid storage function. In striped dolphins, harbor porpoises, and common dolphins increased adipocyte numbers and size and increased lipid content from dorsal to medio-ventral planes in the anterior to mid region of the body have been observed (Koopman, 1998; Tornero et al., 2004; Gómez-Campos et al., 2015). Furthermore, variation of blubber in the anterio-ventral region in harbor porpoises and common dolphins of different nutritional status highlights this region as important to insulation and energy storage (Koopman, 1998; Koopman et al., 2002; Tornero et al., 2004).

The significant differences of AA and AI across girths and planes were also observed among the blubber layers. Significant differences between AA and AI across sampling planes were most pronounced in the middle and inner blubber layers (Figures 6, 7). This may be due to the functional stratification of the blubber in false killer whales as has been observed in other species using histological, lipid quantification, and biochemical methods. Outer layer blubber has been hypothesized to act as a stable mechanical barrier in fin whales and striped dolphins that is less likely to change with physiological state (Aguilar and Borrell, 1991; Gómez-Campos et al., 2015).

In the false killer whale, the middle layer blubber had significant regional differences with larger adipocytes and less connective tissue in the cranial and ventral regions of the body. AI values were consistently small in this layer with less variation than in outer and inner blubber layers. These characteristics suggest that the middle layer blubber may function more as a stable lipid reservoir serving thermoregulatory and buoyancy functions as reported in bottlenose and striped dolphins (Montie et al., 2008; Gómez-Campos et al., 2015).

The inner layer AA and AI values were similar to the middle layer but there was more variability in AI. AA values were intermediate in size compared to the outer and middle layers. This may suggest that the inner layer blubber of the false killer whale is a highly dynamic tissue that changes in response to energy requirements associated with starvation or reproduction as has been observed in several other cetacean species (Aguilar and Borrell 1991; Koopman, 1998; Koopman et al., 2002; Struntz et al., 2004; Montie et al., 2008).

### 4.4 Application

Our findings of large AA, small AI, and higher BT measurements in the cranial and ventral body regions suggest a higher likelihood of detecting changes in this area which is important in assessing nutritional status and overall body condition. However, this presents a challenge for assessments of live, free-ranging cetaceans, as the anterio-ventral region of the body is not easily accessible for sampling. Biopsy sampling is widely utilized for the collection of small skin and blubber samples from live cetaceans, but biopsy sampling targets the dorsolateral plane near the dorsal fin and blubber of incomplete depth. BT measurements are therefore not useful from biopsies as the entire blubber depth is not sampled, specifically the inner layer blubber is often missed, which we anticipate is the most dynamic of the blubber layers. In odontocetes, biopsy depth typically reaches the middle blubber layer which should be preferentially measured for AA and AI metrics over the outer layer when possible. We observed the largest AA in the middle layer, supporting an important role in energy storage, and more pronounced significant differences across sampling regions when compared to the outer layer. When comparing the body location of biopsy sampling to our findings, our results demonstrated that the middle blubber layer AA and AI were not significantly different between the dorsal and dorsolateral plane and only few significant differences were apparent among girth axes from this general region of the body. We anticipate that AA and AI data from the outer or middle layer would be comparable among animals and that the exact location is not needed to provide a representative sample. Prior measures of AA and AI from biopsies collected from southern hemisphere humpback whales demonstrated that it was possible to identify fasting and feeding groups in one study (Castrillon et al., 2017) and sex ratios were correlated with blubber biopsy AA measures in another (Druskat et al., 2019). To our knowledge, AA and AI have not yet been compared in blubber of biopsied odontocetes although AA and AI values were consistent with drone derived body condition assessments in a limited number of pygmy killer whales involved in a prolonged mass stranding event (Currie et al., 2021). Measurement of blubber AA and AI in biopsies has the potential to serve as a valuable tool for conducting overall body condition assessments in odontocetes.

### 4.5 Future investigation

Further work on false killer whales should involve measurements of AA and AI from additional animals that represent different sex, age class, and nutritional status. This is an important step to verifying blubber layers and body regions that can be targeted when attempting to detect changes in body condition among or within individuals. Based on our study, we hypothesize that the anterior and ventral regions, most important to lipid storage, would be different across individuals with varied overall body condition. As this animal was killed by a known fishery interaction it provides valuable baseline data that are representative of a sub-adult male. Similar investigations of additional animals are necessary to confirm that our BT and histology findings are consistent among sub-adults and adults as previous studies have shown that blubber composition and morphology may differ among reproductive status (Lockyer et al., 1985; Koopman, 1998; Konishi, 2006; Gómez-Campos et al., 2015; Roussouw et al., 2022).

Our study was restricted to blubber morphology and histological metrics, but our results demonstrated variation in these blubber characteristics across body regions and by layer. Further investigation into the specific types of lipids, proteins, and metabolites in blubber may provide additional insight into the variability of functionality both among blubber layers and across the body topography (Kershaw et al., 2018). Fatty acid profiles of a variety of species have indicated that the blubber is biochemically stratified. In bottlenose dolphins, polyunsaturated fatty acids deposited in the inner layer are believed to be used for later mobilization whereas the more stable outer layer has a higher concentration of monounsaturated fatty acids, hypothesized to be important for thermoregulation (Samuel and Worthy, 2004; Smith and Worthy, 2006). Considerable investigation has focused on the bottlenose dolphin and other small odontocete species, but limited study of the blubber of false killer whales has been conducted. This is despite the global distribution of the species and the endangered status of an endemic population in Hawai‘i where reduced prey availability and compromised health may represent significant threats.

In conclusion, we established baseline data regarding the variation of blubber morphology and histology across the body of a presumed healthy sub-adult false killer whale. False killer whale blubber was found to be distinctly stratified across blubber depth and non-uniform across body topography. While this study is limited in its inclusion of a single animal, our results are generally in agreement with previously published literature from odontocetes that suggests the inner layer blubber in the anterior-ventral region of the body is the most sensitive to change with nutritional status. We anticipate that blubber histology metrics obtained from biopsy samples is likely to be useful in the evaluation of overall body condition in live false killer whales.

## Data Availability

The raw data supporting the conclusions of this article will be made available by the authors, without undue reservation.

## References

[B1] Ackman R. G. Eaton C. A. Litchfield C. (1971). Composition of wax esters, triglycerides and diacyl glyceryl ethers in the jaw and blubber fats of the Amazon River dolphin (*Inia geoffrensis*). Lipids 6 (2), 69–77. 10.1007/BF02531319 5548640

[B2] Aguilar A. Borrell A. (1991). Heterogeneous distribution of organochlorine contaminants in the blubber of baleen whales: Implications for sampling procedures. Mar. Environ. Res. 31, 275–286. 10.1016/0141-1136(91)90017-3

[B3] Ahima R. S. Flier J. S. (2000). Adipose tissue as an endocrine organ. Trends Endocrinol. Metabolism 11 (8), 327–332. 10.1016/s1043-2760(00)00301-5 10996528

[B4] Baird R. W. (2009). A review of false killer whales in Hawaiian waters: Biology, status, and risk factors. Cascadia research collective. Olympia, WA.

[B5] Bates D. M. (2007). Linear mixed model implementation in Lme4. Manuscript. Madison, WI: Department of Statistics, University of Wisconsin-Madison.

[B6] Bradford A. Baird R. Mahaffy S. Gorgone A. McSweeney D. Cullins T. (2018). Abundance estimates for management of endangered false killer whales in the main Hawaiian Islands. Endanger. Species Res. 36, 297–313. 10.3354/esr00903

[B7] Caon G. Fialho C. B. Danilewicz D. (2007). Body fat condition in franciscanas (*Pontoporia blainvillei*) in Rio Grande do Sul, southern Brazil. J. Mammal. 88, 1335–1341. 10.1644/06-MAMM-A-364R.1

[B8] Castrillon J. Huston W. Nash S. B. (2017). The blubber adipocyte index: A nondestructive biomarker of adiposity in humpback whales (*Megaptera novaeangliae*). Ecol. Evol. 7, 5131–5139. 10.1002/ece3.2913 28770053 PMC5528216

[B9] Castrillon J. Nash S. B. (2020). Evaluating cetacean body condition; A review of traditional approaches and new developments. Ecol. Evol. 10, 6144–6162. 10.1002/ece3.6301 32607220 PMC7319165

[B10] Christiansen F. Vikingsson G. A. Rasmussen M. H. Lusseau D. (2013). Minke whales maximise energy storage on their feeding grounds. J. Exp. Biol. 216, 427–436. 10.1242/jeb.074518 23325860

[B11] Currie J. J. van Aswegen M. Stack S. H. West K. L. Vivier F. Bejder L. (2021). Rapid weight loss in free ranging pygmy killer whales (*Feresa attenuata*) and the implications for anthropogenic disturbance of odontocetes. Sci. Rep. 11, 8181. 10.1038/s41598-021-87514-2 33854117 PMC8046785

[B12] Derous D. ten Doeschate M. Brownlow A. C. Davison N. J. Lusseau D. (2020). Toward new ecologically relevant markers of health for cetaceans. Front. Mar. Sci. 7. 10.3389/fmars.2020.00367

[B13] Doidge D. W. (1990). Integumentary heat loss and blubber distribution in the beluga, *Delphinapterus leucas,* with comparison to the narwal, *Monodon monoceros* . Can. J. Fish. Aquatic Sci. 224, 129–140.

[B14] Druskat A. Ghosh R. Castrillon J. Nash S. M. B. (2019). Sex ratios of migrating southern hemisphere humpback whales: A new sentinel parameter of ecosystem health. Mar. Environ. Res. 151, 104749. 10.1016/j.marenvres.2019.104749 31256980

[B15] Dunkin R. McLellan W. Blum J. Pabst D. (2005). The ontogenetic changes in the thermal properties of blubber from Atlantic bottlenose dolphin *Tursiops truncatus* . J. Exp. Biol. 208, 1469–1480. 10.1242/jeb.01559 15802671

[B16] Evans K. Hindell M. Thiele D. (2003). Body fat and condition in sperm whales, *Physeter macrocephalus*, from southern Australian waters. Comp. Biochem. Physiol. A Mol. Integre. Physiol. 134, 847–862. 10.1016/S1095-6433(03)00045-X 12814793

[B17] Ferreira T. Rasband W. (2012). ImageJ user guide. Bethesda, M.D: IJI.46r. Natl. Inst. Health.

[B18] Galarraga M. Campión J. Muñoz-Barrutia A. Boqué N. Moreno H. Martínez J. A. (2012). Adiposoft: Automated software for the analysis of white adipose tissue cellularity in histological sections. J. Lipid Res. 53, 2791–2796. 10.1194/jlr.D023788 22993232 PMC3494244

[B19] Gearty W. McClain C. R. Payne J. L. (2018). Energetic tradeoffs control the size distribution of aquatic mammals. PNAS 115, 4194–4199. 10.1073/pnas.1712629115 29581289 PMC5910812

[B20] Gómez-Campos E. Borrell A. Correas J. Aguilar A. (2015). Topographical variation in lipid content and morphological structure of the blubber in the striped dolphin. Sci. Mar. 79, 189–197. 10.3989/scimar.04093.25A

[B21] Hamilton J. L. Dillaman R. M. McLellan W. A. Pabst D. A. (2004). Structural fiber reinforcement of keel blubber in harbor porpoise (*Phocoena phocoena*). J. Morphol. 261, 105–117. 10.1002/jmor.10232 15164371

[B22] Irvine L. G. Thums M. Hanson C. E. McMahon C. R. Hindell M. A. (2017). Quantifying the energy stores of capital breeding humpback whales and income breeding sperm whales using historical whaling records. R. Soc. Open Sci. 4, 160290. 10.1098/rsos.160290 28405350 PMC5383807

[B23] Iverson S. J. (2009). “Blubber,” in Encyclopedia of marine mammals. Editors Perrin W. F. Würsing B. M Thewissen J. G. 2nd Edn (New York: Academic Press).

[B24] Kastelein R. A. Helder-Hoek L. Jennings N. (2018). Seasonal changes in food consumption, respiration rate, and body condition of a male harbor porpoise (*Phocoena phocoena*). Aquat. Mamm. 44, 76–91. 10.1578/AM.44.1.2018.76

[B25] Kastelein R. A. Mosterd J. Schooneman N. M. Wiepkema P. R. (2000). Food consumption, growth, body dimensions, and respiration rates of captive false killer whales (*Pseudorca crassidens*). Aquat. Mamm. 26 (1), 33–44.

[B26] Kershaw J. L. Botting C. H. Brownlow A. Hall A. J. (2018). Not just fat: Investigating the proteome of cetacean blubber tissue. Conserv. Physiol. 6, coy003. 10.1093/conphys/coy003 29479430 PMC5814904

[B27] Kershaw J. L. Sherrill M. Davison N. J. Brownlow A. Hall A. J. (2017). Evaluating morphometric and metabolic markers of body condition in a small cetacean, the harbor porpoise (*Phocoena phocoena*). Ecol. Evol. 7, 3494–3506. 10.1002/ece3.2891 28515885 PMC5433969

[B28] Konishi K. (2006). Characteristics of blubber distribution and body condition indicators for Antarctic minke whales (*Balaenoptera bonaerensis*). Mammal. Study 31, 15–22. 10.3106/1348-6160(2006)31[15:COBDAB]2.0.CO;2

[B29] Koopman H. N. Pabst D. A. McLellan W. A. Dillaman R. M. Read A. J. (2002). Changes in blubber distribution and morphology associated with starvation in the harbor porpoise (*Phocoena phocoena*): Evidence for regional differences in blubber structure and function. Physiol. Biochem. Zool. 75, 498–512. 10.1086/342799 12529851

[B30] Koopman H. N. (1998). Topographical distribution of the blubber of harbor porpoises (*Phocoena phocoena*). J. Mammal. 79, 260–270. 10.2307/1382862

[B31] Larrat S. Lair S. (2022). Body condition index in beluga whale (*Delphinapterus leucas*) carcasses derived from morphometric measurements. Mar. Mammal Sci. 38, 274–287. 10.1111/mms.12855

[B32] Lockyer C. (1986). Body fat condition in northeast Atlantic fin whales, *Balaenoptera physalus*, and its relationship with reproduction and food resource. Can. J. Fish. Aquatic Sci. 43, 142–147. 10.1139/f86-015

[B33] Lockyer C. H. McConnell L. C. Waters T. D. (1985). Body condition in terms of anatomical and biochemical assessment of body fat in North Atlantic fin and sei whales. Can. J. Zoology 63, 2328–2338. 10.1139/z85-345

[B34] McClelland S. J. Gay M. Pabst D. A. Dillaman R. Westgate A. J. Koopman H. N. (2012). Microvascular patterns in the blubber of shallow and deep diving odontocetes. J. Morphol. 273, 932–942. 10.1002/jmor.20032 22592863

[B35] Miller C. A. Reeb D. Best P. B. Knowlton A. R. Brown M. W. Moore M. J. (2011). Blubber thickness in right whales *Eubalaena glacialis* and *Eubalaena australis* related with reproduction, life history status and prey abundance. Mar. Ecol. Prog. Ser. 438, 267–283. 10.3354/meps09174

[B36] Montie E. W. Garvin S. R. Fair P. A. Bossart G. D. Mitchum G. B. McFee W. E. (2008). Blubber morphology in wild bottlenose dolphins (*Tursiops truncatus*) from the southeastern United States: Influence of geographic location, age class, and reproductive state. J. Morphol. 269, 496–511. 10.1002/jmor.10602 18157858

[B37] NOAA Fisheries (2021). Endangered species act recovery implementation strategy for the main Hawaiian Islands insular false killer whale (Pseudorca crassidens) distinct population segment. Washington: NOAA Fisheries.

[B38] Noren S. R. Schwarz L. Robeck T. R. (2021). Topographic variations in mobilization of blubber in relation to changes in body mass in short-finned pilot whales (*Globicephala macrorhynchus*). Physiol. Biochem. Zool. 94, 228–240. 10.1086/714637 34010119

[B39] Noren S. R. Udevitz M. S. Triggs L. Paschke J. Oland L. Jay C. V. (2015). Identifying a reliable blubber measurement site to assess body condition in a marine mammal with topographically variable blubber, the Pacific walrus. Mar. Mammal Sci. 31, 658–676. 10.1111/mms.12186

[B40] Norris K. S. (1968). The evolution of acoustic mechanisms in odontocete cetaceans. Evol. Environ. 2, 297–324.

[B41] Odell D. K. Asper E. D. Baucom J. Cornell L. H. (1980). A recurrent mass stranding of the false killer whale, *Pseudorca crassidens,* in Florida. Fish. Bull. 78 (1), 171–177.

[B42] Oleson E. M. Boggs C. H. Forney K. A. Hanson M. B. Kobayashi D. R. Taylor B. L. (2010). “Status review of Hawaiian insular false killer whales (*Pseudorca crassidens*) under the Endangered Species Act,” U.S. Dep. Commer., NOAA Tech. Memo., NOAA-TM-NMFS-PIFSC-22, 140.

[B43] Parlee S. D. Lentz S. I. Mori H. MacDougald O. A. (2014). Quantifying size and number of adipocytes in adipose tissue. Methods Enzym. 537, 93–122. 10.1016/B978-0-12-411619-1.00006-9 PMC4069255 24480343

[B44] Pond C. M. Mattacks C. A. (1985). Body mass and natural diet as determinants of the number and volume of adipocytes in eutherian mammals. J. Morphol. 185 (2), 183–193. 10.1002/jmor.1051850204 29969866

[B45] Raverty S. St. Leger J. Noren D. P. Burek Huntington K. Rotstein D. S. Gulland F. M. D. (2020). Pathology findings and correlation with body condition index in stranded killer whales (*Orcinus orca*) in the northeastern Pacific and Hawai‘i from 2004 to 2013. PloS ONE 15, e0242505. 10.1371/journal.pone.0242505 33264305 PMC7710042

[B46] Read A. J. (1990). Estimation of body condition in harbour porpoises, *Phocoena phocoena* . Can. J. Zoology 68, 1962–1966. 10.1139/z90-276

[B47] Reeves R. R. Leatherwood S. Baird R. W. (2009). Evidence of a possible decline since 1989 in false killer whales (*Pseudorca crassidens*) around the main Hawaiian Islands. Pac. Sci. 63, 253–261. 10.2984/049.063.0207

[B48] Roussouw N. van Vliet T. Naidoo K. Rossouw G. Plön S. (2022). Histomorphological stratification of blubber of three dolphin species from subtropical waters. J. Morphol. 283, 1411–1424. 10.1002/jmor.21511 36059247

[B49] Rstudio Team (2020). Rstudio integrated development for R. Rstudio, pBC. Boston, MA: Rstudio Team.

[B50] Samuel A. Worthy G. (2004). Variability in fatty acid composition of bottlenose dolphin (*Tursiops truncatus*) blubber as a function of body site, season, and reproductive state. Can. J. Zoology 82, 1933–1942. 10.1139/Z05-001

[B51] Schindelin J. Arganda-Carreras I. Frise E. Kaynig V. Longair M. Pietzsch T. (2012). Fiji: An open-source platform for biological-image analysis. Nat. Methods 9, 676–682. 10.1038/nmeth.2019 22743772 PMC3855844

[B52] Shapiro L. G. Stockman G. C. (2001). Computer vision. NJ: Prentice-Hall.

[B53] Smith H. R. Worthy G. (2006). Stratification and intra- and inter-specific differences in fatty acid composition of common dolphin (*Delphinus sp.)* blubber: Implications for dietary analysis. Comp. Biochem. Physiol. B Biochem. Mol. Bio. 143 (4), 486–499. 10.1016/J.CBPB.2005.12.025 16500126

[B54] Struntz D. J. McLellan W. A. Dillaman R. M. Blum J. E. Kucklick J. R. Pabst D. A. (2004). Blubber development in bottlenose dolphins (*Tursiops truncatus*). J. Morphol. 259, 7–20. 10.1002/jmor.10154 14666521

[B55] Tornero V. Borrell A. Forcada J. Pubill E. Aguilar A. (2004). Retinoid and lipid patterns in the blubber of common dolphins (*Delphinus delphis*): Implications for monitoring vitamin A status. Comp. Biochem. Physiol. B Biochem. Mol. Biol. 137, 391–400. 10.1016/j.cbpc.2004.01.001 15050526

[B56] Williams R. Vikingsson G. A. Gislason A. Lockyer C. New L. Thomas L. (2013). Evidence for density-dependent changes in body condition and pregnancy rate of North Atlantic fin whales over four decades of varying environmental conditions. ICES J. Mar. Sci. 70, 1273–1280. 10.1093/icesjms/fst059

